# Inhibiting WNT secretion reduces high bone mass caused by *Sost* loss-of-function or gain-of-function mutations in *Lrp5*

**DOI:** 10.1038/s41413-023-00278-5

**Published:** 2023-08-24

**Authors:** Cassandra R. Diegel, Ina Kramer, Charles Moes, Gabrielle E. Foxa, Mitchell J. McDonald, Zachary B. Madaj, Sabine Guth, Jun Liu, Jennifer L. Harris, Michaela Kneissel, Bart O. Williams

**Affiliations:** 1 Department of Cell Biology, Van Andel Institute, 333 Bostwick Ave., NE, Grand Rapids, MI 49503 USA; 2 Diseases of Aging and Regenerative Medicine, Novartis Institutes for Biomedical Research, CH-4002 Basel, Switzerland; 3 Bioinformatics and Biostatistics Core, Van Andel Institute, 333 Bostwick Ave., NE, Grand Rapids, MI 49503 USA; 4 Oncology, Novartis Institutes for Biomedical Research, San Diego, CA 92121 USA

**Keywords:** Bone, Metabolic disorders

## Abstract

Proper regulation of Wnt signaling is critical for normal bone development and homeostasis. Mutations in several Wnt signaling components, which increase the activity of the pathway in the skeleton, cause high bone mass in human subjects and mouse models. Increased bone mass is often accompanied by severe headaches from increased intracranial pressure, which can lead to fatality and loss of vision or hearing due to the entrapment of cranial nerves. In addition, progressive forehead bossing and mandibular overgrowth occur in almost all subjects. Treatments that would provide symptomatic relief in these subjects are limited. Porcupine-mediated palmitoylation is necessary for Wnt secretion and binding to the frizzled receptor. Chemical inhibition of porcupine is a highly selective method of Wnt signaling inhibition. We treated three different mouse models of high bone mass caused by aberrant Wnt signaling, including homozygosity for loss-of-function in *Sost*, which models sclerosteosis, and two strains of mice carrying different point mutations in *Lrp5* (equivalent to human G171V and A214V), at 3 months of age with porcupine inhibitors for 5–6 weeks. Treatment significantly reduced both trabecular and cortical bone mass in all three models. This demonstrates that porcupine inhibition is potentially therapeutic for symptomatic relief in subjects who suffer from these disorders and further establishes that the continued production of Wnts is necessary for sustaining high bone mass in these models.

## Introduction

Families with inherited mutations in Wnt/beta-catenin signaling have dramatic alterations in bone mass.^1^ In humans, mutations in low-density lipoprotein receptor-related protein 5 (LRP5) lead to overactive Wnt/beta-catenin signaling and enhanced bone formation, causing autosomally dominant high bone mass (HBM). In contrast, loss of LRP5 causes osteoporosis pseudoglioma, an autosomal recessive disorder that causes pediatric osteoporosis.^2^ Gain-of-function missense mutations in *LRP5* that encode proteins incapable of binding the secreted inhibitors DKK1 and sclerostin (SOST) cause HBM in an autosomal dominant manner.^3,4^ Subsequent studies reported that subjects homozygous for loss-of-function mutations in the *SOST* gene developed extreme HBM, a disease known as sclerosteosis.^5,6^ Another HBM disorder, van Buchem disease, was subsequently found to be caused by homozygosity for a 52 kb deletion that removes a *SOST*-specific regulatory element ~35 kb downstream of the *SOST* gene.^7,8^ These observations were the driving force in developing anti-sclerostin antibodies to treat osteoporosis, which culminated in the EU and FDA approval of romosozumab.^9,10^

While subjects with van Buchem disease or HBM associated with *LRP5* mutations have a normal lifespan, they can suffer from neuralgia, headaches, deafness, and facial palsy.^11^ Sclerosteosis subjects have even more severe symptoms that can reduce lifespan. These include cranial vascular and neural foraminal narrowing and reduced intracranial volume, frequent seventh nerve palsy, progressive optic and cranial neuropathies, mixed hearing loss, brainstem compression, intracranial hypertension with increased elastance, and sudden, premature death.^12–15^ Thus, it is critical to identify approaches to reduce the pain and morbidity seen in these subjects to improve their quality of life.

Wnt/beta-catenin signaling is initiated at the plasma membrane via the coordinated action of a frizzled (FZD) receptor and an LRP5 or LRP6 coreceptor following Wnt ligand binding.^16,17^ Extracellular Wnt binding to its cognate receptor and coreceptor initiates intracellular Wnt signaling, often via beta-catenin. Paracrine-acting SOST inhibits Wnt/beta-catenin signaling by binding to LRP5/6 and frizzled coreceptors.^18–20^ Notably, Wnts must be acylated and glycosylated by porcupine (PORCN), an endoplasmic-reticulum resident *O*-acyltransferase, for their secretion.^21–23^ Pharmacological inhibition of PORCN with PORCN inhibitors decreases the growth of Wnt-driven cancers in vivo,^22–26^ supporting their use in cancer clinical trials.^27–29^ However, treatment with PORCN inhibitors or other broad Wnt signaling inhibitors significantly reduces bone mass in both mice and humans, necessitating careful administration of these agents to reduce bone side effects.^27,29–31^

Our previous study demonstrated that a 4-week treatment of PORCN inhibitors in C57BL/6 wild-type (WT) mice substantially reduced bone mass compared to vehicle-only treated animals.^27^ We wanted to extend this work to test whether PORCN inhibitors could reverse the abnormal bone density observed in mice with HBM induced by either loss-of-function mutations in *Sost* or by point mutations in *Lrp5*. To do so, we analyzed two models of human HBM disease. One model was mice with *LRP5* human HBM missense gain-of-function mutations, *LRP5A214V* and *LRP5G171V*, knocked into the endogenous mouse *Lrp5* locus,^32,33^ and the other model was mice with a loss-of-function mutation in *Sost*^34^ to model sclerosteosis. Both mouse models exhibit higher bone strength and material properties, i.e., a HBM phenotype.^32–34^ Mice were treated daily with the potent and selective PORCN inhibitor, LGK974 (WNT974), or a related small-molecule inhibitor with further improved physicochemical properties, GNF-6231,^35^ for 5–6 weeks.

In this study, we investigated whether porcupine inhibitor treatment was sufficient to restore bone mass in HBM mice to WT levels to assess whether using PORCN inhibitors may constitute a potential treatment option to alleviate sclerosing symptoms in HBM subjects with mutations in *SOST* or *LRP5*.

## Results

### Blocking Wnt secretion reduces bone mass and density in *Sost* KO mice

**Fig. 1:**
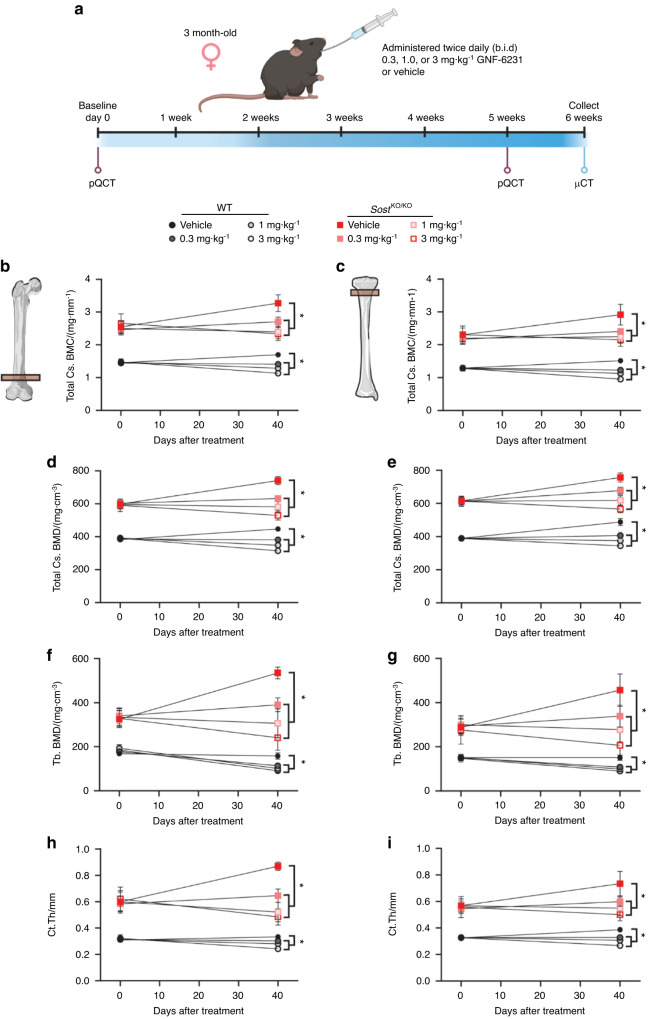
*Sost* treatment schematic and in vivo femoral and tibial pQCT measurements. **a** Schematic diagram of the experimental design. Longitudinal pQCT measurements of distal femur and proximal tibia metaphysis in 3-month-old female wild-type (WT) and *Sost* KO mice treated with vehicle or GNF-6231 daily for 40 days. The parameters measured include (**b**, **c**) total cross-sectional BMC (**d**, **e**) total cross-sectional BMD (**f**, **g**) cancellous BMD, and (**h**, **i**) cross-sectional cortical thickness. For all graphs, the means of each group are indicated by the shape, upper and lower lines represent standard deviations, and *P < 0.05. A minimum of six animals per genotype per condition were analyzed (see Table 1)

**Fig. 2:**
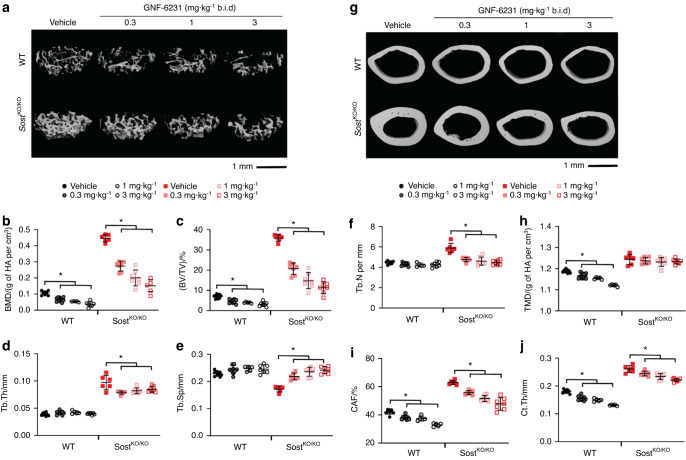
Cancellous and cortical bone analysis of GNF-6231-treated *Sost* KO mice via µCT. Representative images of cancellous bone (**a**) in the distal femur and cortical bone (**g**) in the midshaft of the femur, demonstrating the effects of GNF-6231 treatment on 3-month-old wild-type (WT) and *Sost* KO mice. The trabecular parameters measured included (**b**) bone mineral density (BMD), **c** bone volume/tissue volume (BV/TV), **d** trabecular thickness (Tb.Th), **e** trabecular separation (Tb.Sp), and **f** trabecular number (Tb.N). The cortical parameters measured included (**h**) tissue mineral density (TMD), **i** cortical area fraction (CAF), and **j** cross-sectional thickness (Ct.Th). For all graphs, the means of each group are indicated by the middle line, and the upper and lower lines represent standard deviations. *P < 0.05. A minimum of five animals per genotype per condition were analyzed (Table 1)

We previously demonstrated that female mice harboring a *Sost* loss-of-function mutation show a more pronounced cancellous HBM phenotype than *Sost* loss-of-function male littermates.^34^ To understand whether PORCN inhibition (PORCNi) can reduce HBM and bone architecture defects in *Sost* knockout (KO) mice, we focused on female mice only. We treated adult homozygous *Sost* KO and WT female mice twice a day orally with 0.3, 1, or 3 mg·kg^−1^ GNF-6231 or vehicle control for 6 weeks (Fig. 1a). Peripheral quantitative computed tomography (pQCT) was performed on both the distal femur metaphysis and proximal tibia metaphysis before treatment and at 5 weeks during treatment for longitudinal bone morphology analysis. All doses of PORCNi significantly decreased femoral and tibial total bone mineral content and density, trabecular BMD, and cross-sectional cortical thickness in a dose-dependent manner in WT and *Sost* KO mice as measured by pQCT (Fig. 1b–e). Even at the lowest inhibitor dose, normal late-stage long bone gain was blocked in WT mice. Similarly, elevated bone gain in *Sost* KO animals was reduced at the lowest dose level, and bone loss was induced at the highest dose. Bone gain was similarly suppressed in the cancellous and cortical bone compartments (Fig. 1f–i). Ex vivo microstructural analyses of the femur after the study by high-resolution micro-CT (µCT) confirmed significant reductions in cancellous (from distal femur) and cortical (from femoral midshaft) bone morphometric indices. Dramatic changes in bone mineral density (BMD), bone/tissue volume (BV/TV), trabecular thickness (Tb.Th), trabecular separation (Tb.Sp), and number (Tb.N) were detectable in a dose-dependent manner following PORCNi treatment (Fig. 2). While all treatments reduced the sclerosing bone gain in *Sost* KO female mice, the highest dose of 3 mg·kg^−1^ was best at decreasing both cortical and cancellous bone parameters to levels near those of WT vehicle-treated mice (Fig. 2a–j). At this dose, a 66% reduction in trabecular BMD was observed in *Sost* KO mice (Fig. 2b), and a 68% decrease in bone volume over total tissue volume (BV/TV) (Fig. 2c) relative to vehicle-treated *Sost* KO mice was observed. Tb.Th was decreased by 13%, and Tb.N was reduced by 24%. However, the spacing between trabeculae, Tb.Sp, was increased by 40% compared to *Sost* KO vehicle-treated controls (Fig. 2c–e). These parameters indicate a substantial reduction in the HBM phenotype in sclerostin-deficient bone. However, compared to WT vehicle-treatment, 6 weeks of PORCNi treatment in the *Sost* KO mice was insufficient to completely reverse the HBM phenotype already present at the pretreatment baseline.

Cortical bone morphometric indices were also significantly changed in a dose-dependent manner (Fig. 2g). In *Sost* KO females treated with 3 mg·kg^−1^ of inhibitor, the diaphyseal cortical tissue mineral density (TMD) was not significantly different from that of vehicle-treated *Sost* KO mice (Fig. 2h). However, the cortical bone area fraction (CAF) was decreased by 24%, and the cross-sectional cortical thickness (Ct.Th) was decreased by 15%. This indicates that PORCNi treatment reduces radial bone growth compared to vehicle treatment in *Sost* KO mice without impacting cortical bone quality. Note that a similar change in cortical bone thickness also occurred in WT mice following PORCNi treatment. Thus, PORCN inhibition was sufficient to normalize bone gain in *Sost* KO mice to WT levels during the late-stage long bone growth period.

### Blocking Wnt secretion reverses the HBM observed in *Lrp5* mutants

**Fig. 3:**
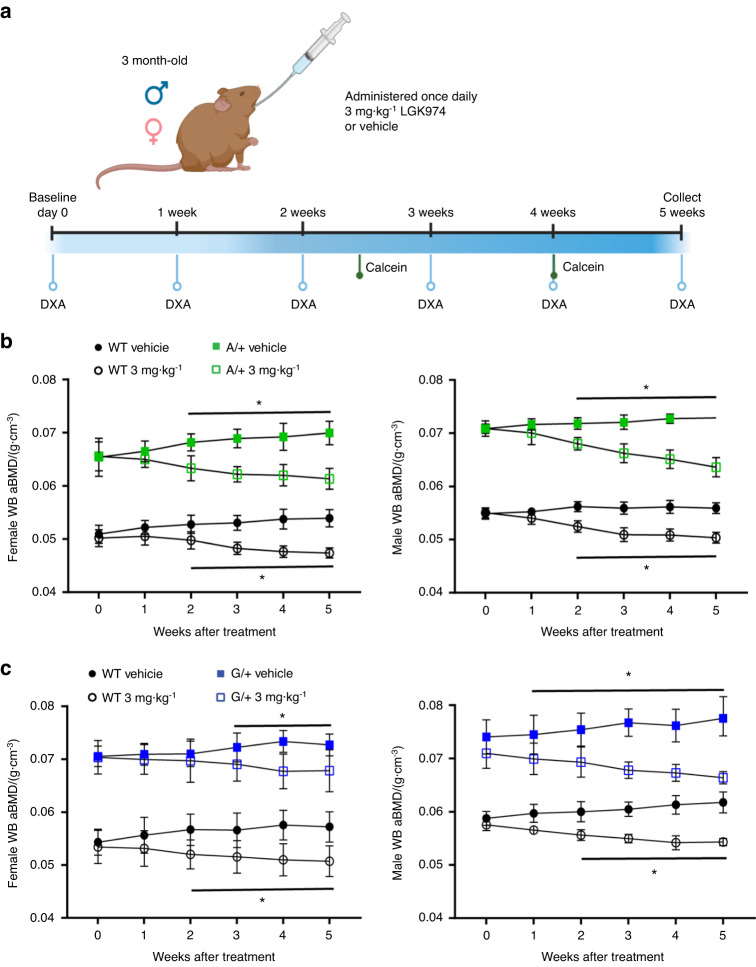
Treatment schematic and whole-body areal bone mineral density measured by DXA. **a** Schematic diagram of the experimental design. **b** Longitudinal whole-body areal bone mineral density (aBMD) measured by DXA of 3-month-old *Lrp5*^*A214V*^ littermate wild-type (WT) and A/+ females and males treated with vehicle or LGK974 daily for 5 weeks. **c** Longitudinal whole-body areal bone mineral density (aBMD), measured by DXA, of *Lrp5*^*G171V*^ littermate wild-type and G/+ females and males treated with vehicle or LGK974 daily for 5 weeks. For all graphs, the means of each group are indicated by the shape. The upper and lower lines represent standard deviations, and *P < 0.05. At least five animals per sex, genotype, and condition were analyzed (see Table 2)

**Fig. 4:**
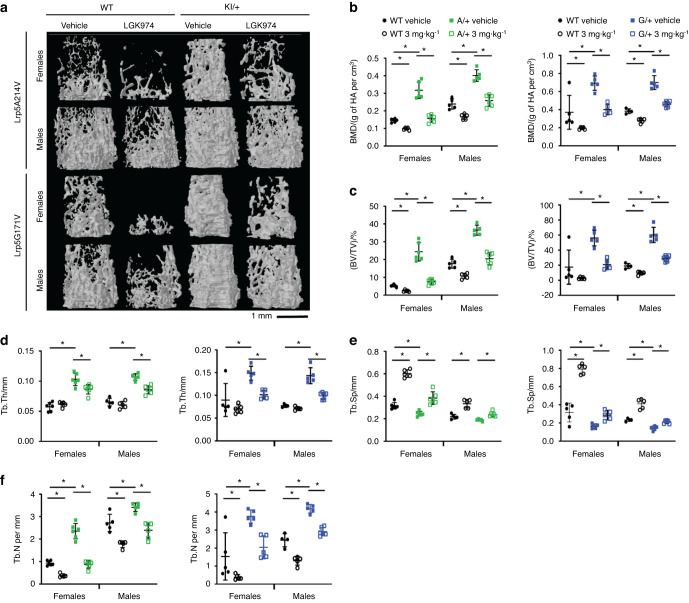
Cancellous bone analysis of LGK974-treated mice via microCT. **a** Representative images of trabecular bone in distal femurs, demonstrating the effects of LGK974 treatment on 3-month-old WT and *Lrp5* mutant mice. The parameters measured included (**b**) bone mineral density (BMD), **c** bone volume/tissue volume, **d** trabecular thickness (Tb.Th), **e** trabecular separation (Tb.Sp), and **f** trabecular number (Tb.N). For all graphs, the means of each group are indicated by the middle line. These upper and lower lines represent standard deviations, and *P < 0.05. A minimum of five animals per sex, genotype, and condition were analyzed (Table 2)

**Fig. 5:**
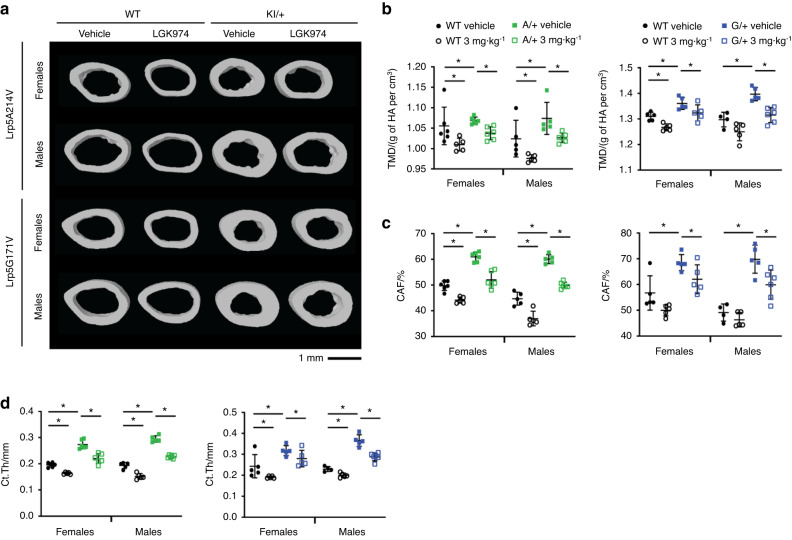
Cortical bone analysis of LGK974-treated mice via µCT. **a** Representative images of cortical bone in the midshaft of femurs, demonstrating the effects of LGK974 treatment on 3-month-old WT and *Lrp5* mutant mice. Trabecular bone parameters of treated *Lrp5A214V* mice, *Lrp5G171V* mice, and their respective controls were measured. These parameters included (**b**) tissue mineral density (TMD), **c** cortical area fraction (CAF), and **d** cross-sectional thickness (Ct.Th). For all graphs, the means of each group are indicated by the middle line, and the upper and lower lines represent standard deviations. *P < 0.05. At least five animals per sex, genotype, and condition were analyzed (Table 2)

We wanted to determine whether PORCNi and, by proxy, the inhibition of Wnt ligand secretion can also reduce the HBM and bone architecture defects in *Lrp5A214V* and *Lrp5G171V* mice. Thus, we treated adult male and female heterozygous mutant mice, hereafter referred to as *Lrp5* A/+ (A/+) and *Lrp5* G/+ (G/+), and littermate WT mice with 3 mg·kg^−1^ LGK974 daily for 5 weeks (Fig. 3a). Because previous studies showed similar increases in bone mass in male and female mice, we included both sexes in these studies. Mice heterozygous for these mutations and WT controls were treated with vehicle or LGK974. Whole-body areal dual-energy X-ray absorptiometry (DXA) scans were collected before treatment and every week thereafter (Fig. 3b, c) to analyze longitudinal BMD changes. After 5 weeks of treatment, femurs were collected, and µCT and histomorphometric analyses were performed. We quantified trabecular bone in the distal femur (Fig. 4) and cortical bone in the femoral midshaft (Fig. 5).

Longitudinal analysis of the whole-body areal BMD (aBMD) by DXA gave us insight into the effects of LGK974 on overall bone architecture in both WT and *Lrp5* mutant heterozygotes. Note that aBMD was significantly higher in vehicle-treated *Lrp5* A/+ and *Lrp5* G/+ male and female mice than in their vehicle-treated WT littermates (Fig. 3b, c). aBMD significantly decreased in both female and male WT and A/+ treated animals starting 2 weeks after treatment (Fig. 3b), whereas *Lrp5* G*/+* animals responded differently to LGK974 treatment. In female *Lrp5* G*/+* mice, significant aBMD changes were first observed after 3 weeks of treatment (Fig. 3c). In contrast, male *Lrp5* G*/+* mice had minor but significant aBMD changes starting after the first week of treatment (Fig. 3c).

**Fig. 6:**
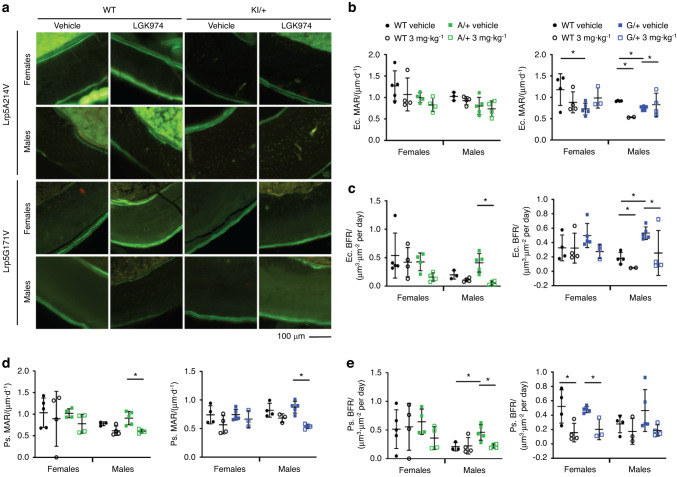
Dynamic histomorphometry of femoral cortical bone. **a** Representative images of cortical cross-sections from fluorochrome-labeled femurs. **b** The endocortical mineral apposition rate (Ec.MAR), **c** endocortical bone formation rate (Ec.BFR), **d** periosteal mineral apposition rate (Ps.MAR), and **e** periosteal bone formation rate (Ps.BFR). For all graphs, the means of each group are indicated by the middle line, and the upper and lower lines represent standard deviations. *P < 0.05. At least three animals per sex, genotype, and condition were analyzed (Table 2)

Bone ultrastructure and density were consistently and significantly elevated in vehicle-treated *Lrp5* A/+ and *Lrp5* G/+ mice for cancellous (Figs. 3 and 4) and cortical bone (Figs. 5 and 6) density parameters, in support of earlier work.^32,33^ As previously observed,^33^ average trabecular BMD was dramatically increased in vehicle-treated *Lrp5* A*/+* and *Lrp5* G*/+* mice compared to their vehicle-treated WT littermates (Fig. 4b). The cancellous bone framework was decreased in both sexes because of LGK974 treatment (Fig. 4a), although female mice had a more significant response to LGK974 treatment. Treatment with LGK974 significantly decreased BMD to levels very close to those observed in WT vehicle-treated mice in both female *Lrp5* A*/+* and *Lrp5* G*/+* mice (Fig. 4b). LGK974 treatment also significantly decreased BV/TV in both sexes of *Lrp5* A*/+* and *Lrp5* G*/+* mice compared to their vehicle-treated WT littermates (Fig. 4c). Moreover, Tb.Th and Tb.N in both LGK974-treated models decreased to levels closer to those in vehicle-treated WT mice (Fig. 4d, f). Similar to PORCNi treatment of *Sost* KO mice (Fig. 2), LGK974 treatment increased Tb.Sp in all conditions (Fig. 4e). These parameters also show that treating *Lrp5* HBM models with porcupine inhibitors can substantially reduce the HBM phenotype.

To test whether LGK974 treatment could normalize the altered bone architecture of *Lrp5* mutant mice, we also quantified cortical bone density and structure (Fig. 5). The increase in cortical TMD, CAF, and bone cross-sectional cortical thickness (Ct.Th) observed in *Lrp5* mutant variants was significantly reduced following LGK974 treatment (Fig. 5b–d). All these parameters demonstrate that the cortical bone architecture became normalized compared to WT vehicle-treated littermates.

Similar to the *Sost* KO model, µCT analysis revealed that LGK974 successfully normalized the HBM phenotypes in *Lrp5* A*/+* and *Lrp5* G*/+* mice. When vehicle-treated WT mice were compared to LGK974-treated *Lrp5* A*/+* and *Lrp5* G*/+* mice, the cancellous and cortical bone parameters were very similar. Thus, the HBM effects driven by *Lrp5* A*/+* and *Lrp5* G*/+* mutations were mitigated with PORCNi to normalize bone volume and architecture.

### Blocking Wnt ligand secretion reduces periosteal and endocortical bone formation in *Lrp5* mutants

To evaluate bone remodeling in our experiments, we employed dynamic histomorphometry using the mineral apposition rate (MAR) and bone formation rate (BFR) to assess the cellular nature of the bone changes. Cortical bone was fluorochrome-labeled via calcein injections 10 days apart (at 17 days and 7 days before sacrifice; Fig. 3a). While no significant decrease in the endocortical mineral apposition rate (Ec.MAR) was observed in vehicle- or LGK974-treated *Lrp5* A*/+* mice, the Ec.MAR was significantly decreased in vehicle-treated *Lrp5* G*/+* mice compared to vehicle-treated WT mice (Fig. 6b). In male *Lrp5* G*/+* mice, treatment with LGK974 increased the Ec.MAR. The endocortical bone formation rate (Ec.BFR) trended upward in vehicle-treated male *Lrp5* A*/+* mice and was significantly elevated in *Lrp5* G*/+* mice compared to the WT vehicle group (Fig. 6c), suggesting increased bone remodeling in HBM males. This difference was not observed between vehicle-treated WT and mutant female mice (Fig. 6c). LGK974 treatment led to a significant decrease in the Ec.BFR for male *Lrp5* A*/+* and *Lrp5* G*/+* mice compared to vehicle-treated *Lrp5* mutant mice (Fig. 6c). With our sample size, we observed a significant difference in bone dynamics in female mice from either *Lrp5* mutant mice treated with LGK974. The periosteal mineral apposition rate (Ps.MAR) in both A*/+* and G*/+* males treated with LGK974 was significantly decreased compared to that in vehicle-treated mutant mice (Fig. 6d). Additionally, male *Lrp5* A*/+* mice and WT littermate control mice treated with LGK974 had a statistically significant decrease in periosteal bone formation (Ps.BFR) (Fig. 6e). The only significant difference in cortical dynamics for female mice following LGK974 treatment was observed in *Lrp5* G*/+* mice, where Ps.BFR was significantly reduced by LGK974 (Fig. 6e). However, male mice were not significantly affected. In conclusion, endocortical and periosteal bone formation decreased when *Lrp5* HBM mutants were treated with LGK974.

### Cellular bone changes are rescued following LGK974 treatment in *Lrp5* mutant mice

**Fig. 7:**
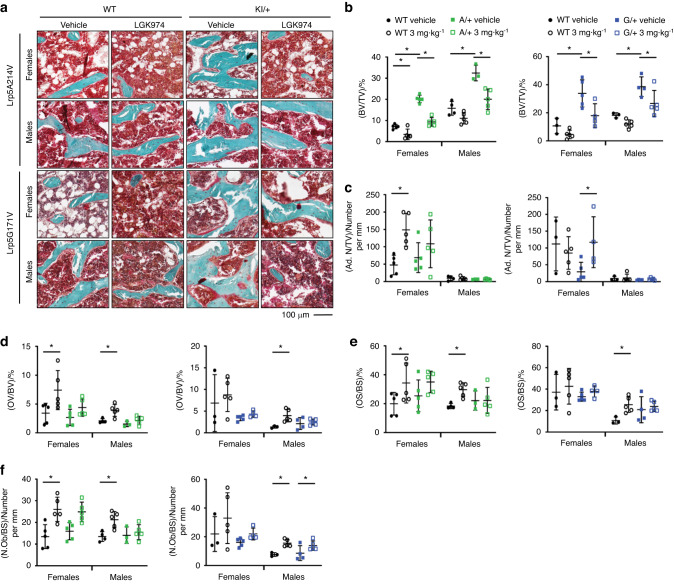
Static histomorphometry of femoral trabecular bone. **a** Representative images of Goldner’s trichrome-stained trabecular bone of the distal femurs of mice. **b** The bone volume/tissue volume (BV/TV), **c** adipocyte number/tissue volume (Ad. N/TV), **d** osteoid volume/bone volume (OV/BV), **e** osteoid surface/bone surface (OS/BS), and **f** number of osteoblasts/bone surface (N.Ob/BS). For all graphs, the means of each group are indicated by the middle line, and the upper and lower lines represent standard deviations. *P < 0.05. At least three animals per sex, genotype, and condition were analyzed (Table 2)

**Fig. 8:**
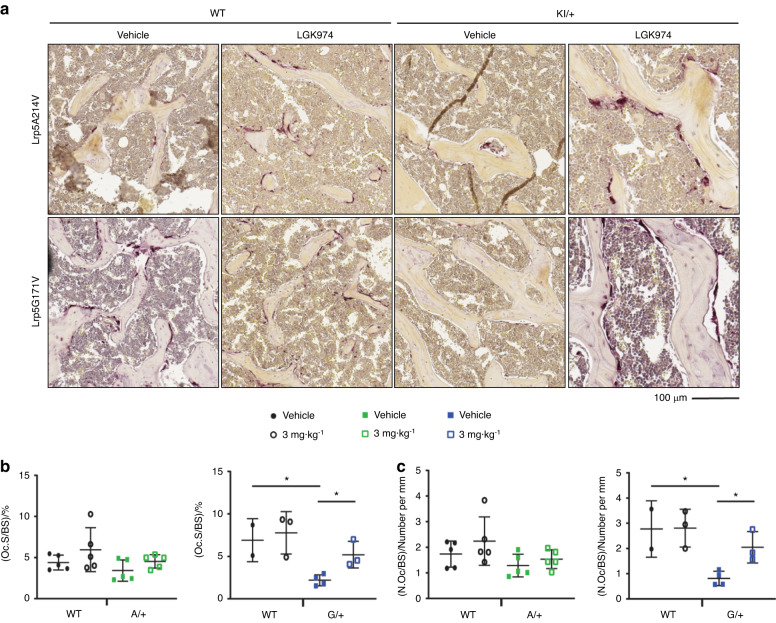
Osteoclast quantification in distal femurs of mice. **a** Representative images of TRAP-stained femoral sagittal sections, **b** osteoclast surface/bone surface (Oc.S/BS), and **c** number of osteoclasts/bone surface (N.Oc/BS). For all graphs, the means of each group are indicated by the middle line, and the upper and lower lines represent standard deviations. *P < 0.05. A minimum of two animals per genotype and per condition were analyzed

To investigate how PORCNi impacts the skeleton of *Lrp5* mutant mice at the cellular level, we stained sections of cancellous bone with Goldner’s trichrome (Fig. 7a) and tartrate-resistant acid phosphatase (TRAP) (Fig. 8). The trends for increased bone volume obtained by µCT were confirmed in the cancellous bone sections (Fig. 7b). The BV/TV was significantly increased in both sexes of vehicle-treated *Lrp5* A*/+* and *Lrp5* G*/+* mice compared to their WT controls (Fig. 7b). Furthermore, Goldner’s trichrome staining showed changes in the number of adipocytes within females, specifically WT (*Lrp5A214V* littermates) and *Lrp5* G*/+* mice treated with LGK974 (Fig. 7c). Osteoid volume/bone volume (OV/BV) also significantly increased with the addition of LGK974 in WT mice from the *Lrp5* A*/+* and *Lrp5* G*/+* groups (Fig. 7d). Likewise, the same groups displayed a significant increase in osteoid surface/bone surface (OS/BS). A significant increase was observed in WT mice when treated with LGK974 (littermates of the *Lrp5* A*/+* mice: 72% change in females and 61% in males; littermates *of Lrp5* G*/*+: 136% change in males only) (Fig. 7e). However, no significant differences were observed in either vehicle- or LGK974-treated *Lrp5* mutant mice (Fig. 7e). A significant increase in the number of osteoblasts/bone surface (N.Ob/BS) occurred between vehicle- and LGK974-treated WT mice (Fig. 7f) in both females (by 93.5%) and males (by 57.6%). Within the *Lrp5* G*/+* group, treatment with LGK974 significantly increased N.Ob/BS in both WT and *Lrp5* G*/+* male mice (Fig. 7f).

We analyzed TRAP-stained sections of trabecular bone from male mice to investigate changes in osteoclast function in *Lrp5* HBM mutants and LGK974-treated animals. Osteoclast surface/bone surface (Oc.S/BS) was significantly decreased in vehicle-treated *Lrp5* G*/+* mice compared to vehicle-treated WT mice (Fig. 8b). When these Lrp5 G/+ mice were treated with LGK974, Oc.S/BS was significantly increased compared to the vehicle-treated mutant mice (Fig. 8b). However, there was no significant change in Oc.S/BS in vehicle- or LGK974-treated *Lrp5* A*/+* mice. (Fig. 8b). The number of osteoclasts/bone surface (N.Oc/BS) was significantly decreased in vehicle-treated *Lrp5* G*/+* mice (Fig. 8c). Additionally, when the *Lrp5* G*/+* mice were treated with LGK974, the N.Oc/BS increased significantly (Fig. 8c). Again, there was no significant change in the N.Oc/BS in vehicle- or LGK974-treated *Lrp5* A*/+* mice (Fig. 8c).

## Discussion

Seminal work performed over 20 years ago established the critical role of Wnt signaling in bone homeostasis. Homozygosity for loss-of-function mutations in *LRP5* was identified as the underlying cause of osteoporosis pseudoglioma,^2^ a syndrome characterized partly by early-onset, severe osteoporosis. Almost concurrently, subjects carrying autosomal dominant point mutations in *LRP5* were shown to develop high bone mass.^3,4^ These point mutants produced an altered form of LRP5 that could no longer be bound and inhibited by negative regulators of the pathway, such as dickkopf-1 and sclerostin.^4,18,19,36,37^ Subsequent work showed that subjects either completely lacking *SOST* expression (sclerosteosis^6,38^) or having severely diminished *SOST* expression due to loss of a regulatory enhancer sequence that acted on the *SOST* promoter (van Buchem disease^39^) also developed extremely high bone mass. These observations were the basis for the biotechnology industry investing in developing therapeutic antibodies that blocked SOST activity, which ultimately resulted in successfully developing an approved therapeutic, romosozumab, to treat severe osteoporosis.^40–42^ While this may benefit thousands of subjects with osteoporosis in the future, subjects with high bone mass disorders (and those found to have mutations in *LRP6*^43,44^ analogous to those with *LRP5* point mutations) continue to suffer from the secondary effects of bone overgrowth, such as increased intracranial pressure, loss of vision and hearing, and progressive overgrowth of other craniofacial structures. We sought to test whether selectively inhibiting Wnt signaling could lessen these symptoms using well-validated mouse models of these HBM disorders.

Porcupine is a membrane-bound *O*-acyltransferase with highly specific activity directed at adding a palmitoleic acid hydrocarbon chain at a conserved serine residue in all Wnts.^21,45,46^ Given the increased Wnt signaling activity associated with many human tumors,^47,48^ porcupine inhibitors were developed as potential treatments for cancer subjects. While these showed significant activity against tumor growth, phase 1 clinical trials with porcupine inhibitors were paused due to increased risks of bone fracture associated with decreased bone mass.^27,28,49^ Consistent with this observation, genetically engineered mouse models with osteoblast-specific inactivating mutations in porcupine or the dedicated Wnt chaperone *GPR177* (wntless) have low bone mass.^50–52^ We previously showed that rapid bone loss was caused by treating WT mice with porcupine inhibitors.^27^ Our current work further extends this concept by showing bone loss in high-bone-mass disorders caused by mutations in Wnt signaling pathway components.

Our studies were designed and powered to evaluate changes in long-bone skeletal growth. Furthermore, at this age and for this duration of treatment, long bones are growing faster than cranial structures, and it may be easier to discern changes in bone parameters. However, given the neurological symptoms in human HBM patients, changes in cranial bone parameters are also of obvious interest. To evaluate this, we performed DXA on a similar cranial region from the same control and *Lrp5* A/+ and *Lrp5* G/+ animals at the beginning and end of treatment (Fig. S1). Treatment with the porcupine inhibitor reduced BMD in wild-type male and female mice over these 4 weeks, while untreated wild-type mice had stable cranial BMD. All Lrp5 A/+ or *Lrp5* G/+ animals exposed to the control (vehicle-only) injections exhibited increases in cranial BMD during the study period. In contrast, all *Lrp5* A/+ or *Lrp5* G/+ males and females exposed to the porcupine inhibitor exhibited reductions in BMD in this same context. Thus, this work is consistent with the idea that porcupine inhibitors reduce cranial BMD.

Our work indicates that the effects of *Sost* and *Lrp5* mutations in causing HBM can be normalized by blocking Wnt ligand secretion. This is consistent with previous work showing that HBM-associated variants were not intrinsically more active than wild-type variants but were no longer inhibited by endogenous Wnt inhibitors.^4,19,36,37^ This further supports the notion that the maintenance of HBM associated with *Lrp5A214V* and *Lrp5G171V* mutations and loss of function of *Sost* is dependent on the continued presence of Wnt ligands, validating our earlier in vitro^36^ and in vivo observations.^27^

Our work also reinforces that inhibiting Wnt signaling with porcupine inhibitors increases osteoclast activity.^27^ One mechanistic explanation for this would be that osteoprotegerin is activated by Wnt/beta-catenin signaling.^53,54^ Thus, inhibition of Wnt activity would lead to decreases in OPG and increases in osteoclast activity. Our earlier work also showed that combining porcupine inhibition with agents that block bone resorption (i.e., bisphosphonates) could prevent bone loss^27^ and allow for the use of porcupine inhibitors in phase 1 clinical trials for cancer treatment without severe skeletal side effects.

Subjects with sclerosteosis, van Buchem disease, or HBM associated with *LRP5* mutations often suffer from neuropathies and other sequelae due to nerve impingements caused by increased bone mass.^11–15^ We show here that when treated with Wnt secretion inhibitors, genetic mouse models that replicate human HBM exhibit reduced levels of bone mass, and these levels are similar to normal levels. Therefore, this approach could serve as a potential strategy for treating HBM subjects by reducing neuropathy. This concept is supported by the observed loss in bone mass associated with the clinical use of PORCN inhibitors.^27^ Our work shows that a 4- to 6-week treatment regimen is sufficient to reduce bone mass. We expect similar, or even shorter, treatments with PORCN inhibitors will be sufficient to provide symptomatic relief for neuropathies. This work is the conceptual foundation for conducting longitudinal studies to further define the treatment frequency needed to mitigate HBM-related neuropathies. While we did not note any grossly observable deleterious effects of Porcupine inhibition in these studies, longer-term effects must be carefully evaluated. The benefits of managing skeletal-related symptoms with porcupine inhibitors must be balanced with the potential side effects of reducing porcupine activity in other tissues.^55,56^ Other options are available for treating HBM patients by blocking Wnt signaling to alleviate their symptoms. These include using a recombinant sclerostin-Fc fusion protein, which has been effective in mouse models.^57^ In addition, several other strategies for blocking Wnt signaling in vivo are in development and would be potentially applicable to the symptomatic management of HBM patients.^58^

## Materials and methods

### Animals

Mice with *Lrp5* conditional knock-in HBM alleles (p.G171V and p.A214V) were previously described.^32,33^ Animals used in this study were initially crossed to a ubiquitous Cre (CMV-Cre) transgenic mouse to excise the neomycin-resistant cassette flanked by *loxP* sites and then backcrossed to C57BL/6J animals to breed out the Cre transgene. We used WT (+/+) littermate controls and heterozygous Lrp5-A214V (A/+) or Lrp5-G171V (G/+) mice, as previously described.^2^ WT female and male 3-month-old and heterozygous *Lrp5-A214V* (A/+) or *Lrp5-G171V* (G/+) mice were treated daily with either 3 mg·kg^−1^ LGK974 (provided by Novartis) or with vehicle for 5 weeks by oral gavage at a dosing volume of 10 μL·g^−1^ animal body weight. LGK974 has poor solubility and is therefore administered as a suspension in 0.5% methylcellulose/0.5% Tween 80.^23,59^ *Lrp5* HBM mice were maintained following institutional animal care and use guidelines, and experimental protocols were approved by the Institutional Animal Care and Use Committee of the Van Andel Institute. Mice were housed in Thoren Maxi-Miser IVC caging systems with a 12-h/12-h light/dark cycle and fed a breeder rodent diet containing 23% protein and 24% fat with an energy content of 19.3 MJ·kg^−1^ (5021, LabDiet St. Louis MO), with food and water provided ad libitum.

Three-month-old nonlittermate WT female (C57BL/6J, Charles River Laboratories, Germany) and *Sost* KO mice,^34^ which had been fully backcrossed to the C57BL/6J genetic background, were treated twice daily for 6 weeks with either vehicle (0.5% methylcellulose/0.5% Tween 80 in water) or a GNF-6231 (provided by Novartis) suspension at 0.3, 1 or 3 mg·kg^−1^ by oral gavage at a dosing volume of 10 μL·g^−1^ animal body weight. Mice were housed at 22 °C under a 12-h/12-h light/dark cycle and were fed a standard rodent diet containing 18.2% protein and 3.0% fat with an energy content of 15.8 MJ·kg^−1^ (3890, Provimi Kliba SA), with food and water provided ad libitum. Procedures with *Sost* mice conformed to Swiss federal law for animal protection controlled by the Basel‐Stadt Cantonal Veterinary Office, Switzerland.

For dynamic histomorphometry analysis of *Lrp5* HBM models, animals were administered the fluorescent dye calcein (10 mg·kg^−1^ split equally between i.p. and sub.q.; MilliporeSigma) 17 and 7 days before euthanasia. All animals were euthanized at 17 weeks of age, following 5 weeks of treatment. Femurs were isolated and fixed in 10% neutral-buffered formalin (NBF) at room temperature for 48 h and then changed to 70% ethanol before analysis and histological processing.

Tables 1, 2 details the number of specimens analyzed for each application.

**Table 1:** Number of *Sost* animals analyzed per genotype and treatment condition

Analysis	WT				*Sost*^*−/−*^			
	Vehicle	0.3 mg·kg^−1^	1 mg·kg^−1^	3 mg·kg^−1^	Vehicle	0.3 mg·kg^−1^	1 mg·kg^−1^	3 mg·kg^−1^
µCT	10	9	5	7	6	7	7	7
pQCT	10	9	10	9	6	7	7	7

**Table 2:** Number of *Lrp5* animals analyzed per genotype and treatment condition

Analysis	Vehicle treatment								LGK974 treatment							
	*A214V* +/+ female	A/+ female	*A214V* +/+ male	A/+ male	*G17V* +/+ female	G/+ female	*G17V* +/+ male	G/+ male	*A214V* +/+ female	A/+ female	*A214V* +/+ male	A/+ male	*G17V* +/+ female	G/+ female	*G17V* +/+ male	G/+ male
DXA and µCT	6	6	5	5	5	5	5	5	5	6	5	6	5	5	5	6
Dynamic histomorp-hometry	5	4	3	5	4	5	4	5	4	4	4	4	4	3	3	3
Static histomorp-hometry	5	5	4	3	3	5	3	4	5	5	5	5	5	4	5	5
TRAP			5	5			2	4			5	5			3	3

### DXA

We performed whole-body dual-energy X-ray absorptiometry (DXA) to measure areal bone mineral density (aBMD; gm·cm^−2^) and bone mineral content (BMC; gm) for the postcranial skeleton as well as the cranium. For cranium measurements, we analyzed the parietal, interparietal and occipital bones of the skull. A PIXImus II bone densitometer (GE Lunar) was used for analysis. aBMD values were collected at 0, 1, 2, 3, 4, and 5 weeks into treatment.

### Microcomputed tomography (µCT)

The *Lrp5* HBM models were analyzed using a SkyScan 1172 µCT system (Bruker MicroCT: Kontich, Belgium). Femora were scanned in 70% ethanol using an X-ray voltage of 60 kV, current of 167 µA, and 0.5 mm aluminum filter with a voxel size of 7.98 µm. Femoral images were reconstructed using NRecon 1.7.4.6 (Bruker MicroCT). The mineralized tissue was oriented, and a volume of interest (VOI) was defined using DataViewer 1.5.6.3 (Bruker MicroCT). Regions of interest (ROIs) were defined for cortical and trabecular bone using CTAn 1.18.8.0 (Bruker MicroCT). A trabecular ROI was drawn in the distal epiphysis for each femur, beginning 0.25 mm proximal to the growth plate and 2.5 mm in height. To define each position of the cortical ROI, the distal end of the region was set to 45% of the femur length. The ROI was 0.8 mm in height toward the proximal end of the bone, within the midshaft. Trabecular 3D analysis was performed to quantify bone mineral density (BMD), bone volume/tissue volume (BV/TV), bone surface/bone volume, trabecular thickness (Tb.Th), trabecular separation, (Tb.Sp), and trabecular number (Tb.N). Cortical 2D analysis was performed to quantify tissue mineral density (TMD), tissue area, bone area, cortical area fraction (bone area/tissue area, CAF), cross-sectional thickness, and bone perimeter.

Femora from *Sost* KO mice were analyzed by high-resolution ex vivo μCT using a vivaCT 40 instrument (Scanco Medical AG) at a voxel dimension of 6 μm. A Gaussian filter (*σ* = 0.7, support of one voxel) was used in all analyses to suppress noise, and a segmentation threshold of 275 was applied.

### Peripheral quantitative computed tomography (pQCT)

Treatment efficacy was assessed longitudinally by in vivo pQCT under isoflurane inhalation using an adapted Stratec‐Norland XCT‐2000 instrument fitted with an Oxford (Oxford, UK) 50-mm X‐ray tube (GTA6505M/LA) and a 0.5‐mm‐diameter collimator (voxel size: 0.07 mm × 0.07 mm × 0.4 mm).

### Static and dynamic histomorphometry

Detailed static and dynamic histomorphometry tissue preparation and analysis methods are found in our published protocol for skeletal tissue phenotyping.^60^

Briefly, fixed fluorochrome-labeled femurs were dehydrated in graded ethanol and cleared using xylene. Samples were infiltrated and embedded in plastic using 85%–100% MMA and 15% dibutyl phthalate until polymerization. Femurs were sectioned coronally, deplasticized, Goldner’s trichrome stained, and coverslipped for static histomorphometry.

Additional femurs were fixed and decalcified in 10% EDTA for 14 days, embedded in paraffin, and sectioned sagittally. Sections were stained with hematoxylin and eosin (H&E) or tartrate-resistant acid phosphatase (TRAP kit 387A; MilliporeSigma, St. Louis, MO, USA). Indices measured for Goldner’s trichrome-stained slides included bone volume/tissue volume (BV/TV), adipocyte number/tissue volume (Ad.V/TV), osteoid volume/bone volume (OV/BV), osteoid surface/bone surface (OS/BS), and the number of osteoblasts/bone surface (N.Ob/BS). Parameters measured for TRAP-stained slides included osteoclast surface/bone surface and the number of osteoclasts/bone surface. All histomorphometric analyses were performed using BIOQUANT OSTEO software (v19.2.60; BIOQUANT Image Analysis Corporation, Nashville, USA).

For dynamic histomorphometry, femoral midshafts were cross-sectioned and placed in a coverslip. The endocortical mineral apposition rate (MAR), endocortical bone formation rate (BFR), periosteal MAR, and periosteal BFR were measured for each sample.

### Statistical analyses

All analyses, except DXA, were performed using the same methods. All percentage data, including BV/TV (µCT), CAF, BV/TV (histomorphometry), OV/BV, OS/BS, and Oc.S/BS were analyzed via beta regression. All other outcomes were analyzed via robust linear regression with natural log (*y* + 0.01) transformation to improve the fit of the model. All analyses and outcomes were Benjamini‒Hochberg false discovery rate (FDR) adjusted to account for multiple testing. To determine if the two groups were similar, an equivalence test was performed such that any nonsignificant difference with a 95% confidence interval entirely within the range of 0.9–1.1 (fold-change [FC]) was considered equivalent. For any differences we could confidently say are smaller than 10%, we can conclude that there is sufficient evidence to support a hypothesis that there is no difference between the groups.

DXA data were analyzed using linear mixed-effects models with random slopes and intercepts for each animal. FDR-adjusted linear contrasts were used to test specific hypotheses. Analyses were stratified by sex. A three-way interaction between weeks, genotype, and treatment was initially fit for all models. All two-way interactions were also tested and dropped if *P* values exceeded 0.15 and saved for the interaction between weeks and treatment. This allowed for testing of a treatment effect in all comparisons.

### Supplementary information

Supplementary Figure S1

## Data Availability

The data analyzed to support the findings in this study are available from the corresponding author upon request.
