# Supplementary material for: Inhibiting WNT secretion reduces high bone mass caused by Sost loss-of-function or gain-of-function mutations in Lrp5
Source: Bone Res. 2023 Aug 24;11:47. doi: 10.1038/s41413-023-00278-5 (PMC10447437; doi:10.1038/s41413-023-00278-5)
Supplement: Supplementary file 1 — Supplementary Figure S1 [file 41413_2023_278_MOESM1_ESM.docx]

**Figure S1. Areal skull bone mineral density of LGK974-treated mice measured by DXA**. (a) Longitudinal aBMD of the skull, measured by DXA, of 3-month-old *Lrp5^A214V^* littermate wild-type (WT) and A/+ females and males treated with vehicle or LGK974 daily for 5 weeks. (b) Longitudinal aBMD of the skull, measured by DXA, of *Lrp5^G171V^* littermate wild-type and G/+ females and males treated with vehicle or LGK974 daily for 5 weeks. For all graphs, the means of each group are indicated by the shape, and the upper and lower lines represent standard deviations. * = p < 0.05. A minimum of 4 animals per genotype per condition were analyzed.

**Supplemental Figure 1**

**
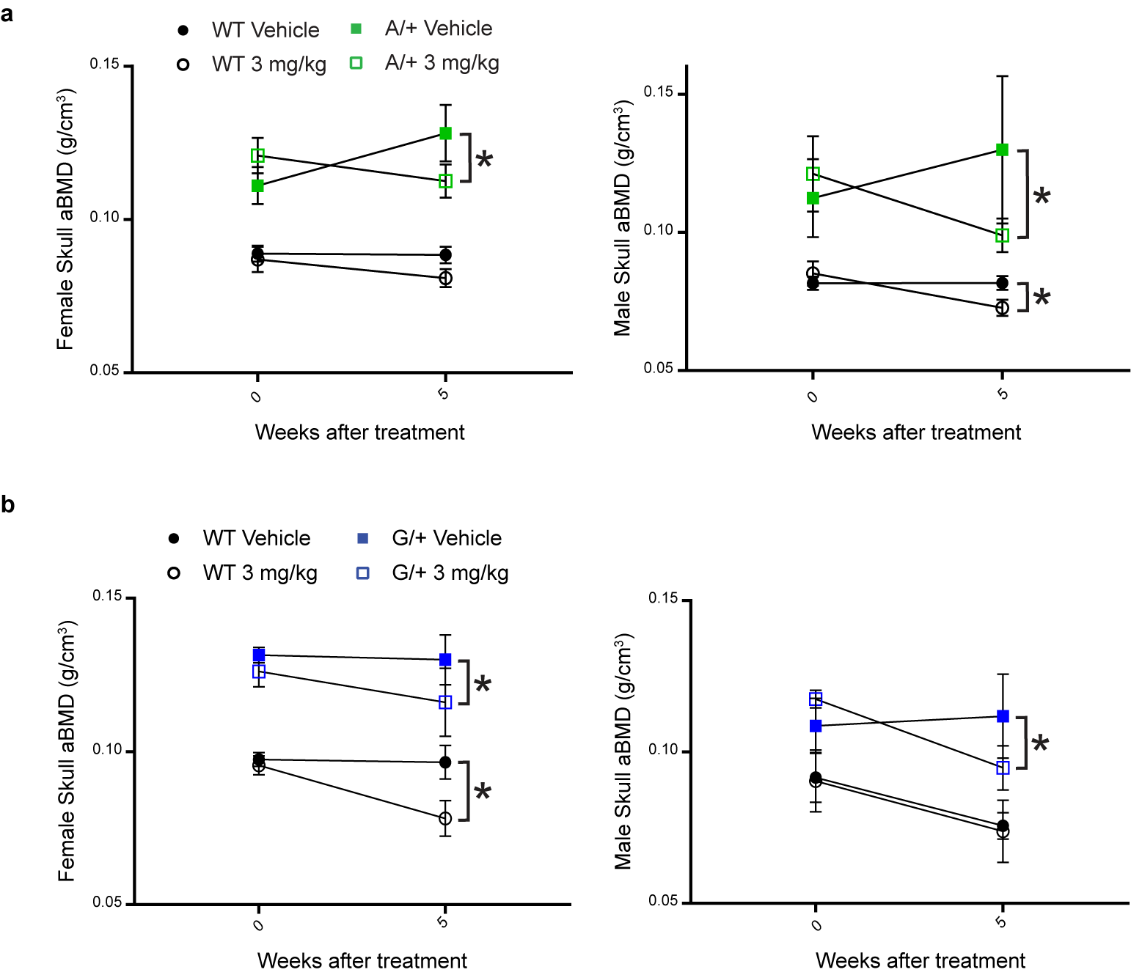
**
